# Neuropsychological and internalizing problems in acute central nervous system infections: a 1 year follow-up

**DOI:** 10.1186/s13052-017-0416-2

**Published:** 2017-10-24

**Authors:** Bozzola Elena, Bergonzini Paola, Bozzola Mauro, Tozzi Alberto Eugenio, Masci Marco, Rossetti Chiara, Carloni Emanuela, Villani Alberto

**Affiliations:** 1 Pediatric and Infectious Diseases Unit, IRCCS Bambino Gesù Children Hospital, Rome, Italy; 2 Internal Medicine and Therapeutics Department, Pediatrics and Adolescentology Unit, University of Pavia, Fondazione IRCCS San Matteo, Pavia, Italy; 3 Sanitary Direction, IRCCS Bambino Gesù Children Hospital, Rome, Italy

**Keywords:** Central nervous system infection, Neuropsychology, Psychological test

## Abstract

**Background:**

Acute central nervous system (ACNS) infections such as meningitis, encephalitis and cerebellitis still cause morbidity and mortality among children. The aim of this study was to verify whether neuropsychological impairment may develop in ACNS survivors.

**Methods:**

The study included pediatric patients affected by ACNS disorders, aged 3-16 years admitted to the Bambino Gesù Children Hospital, Rome from June 2013 till June 2015. The patients and their parents underwent a psychological interview and neuropsychological tests during the first week of hospital admission and 1 year after, during a follow-up control. Wilcoxon signed rank tests for paired data were conducted to verify if the results were statistically importance. Patients underwent a cognitive profile test through the Leiter international performance scale – revised, motor skills evaluation through the test of visual-motor integration and a psychopathological evaluation by the child behavior checklist. The K-SADS-PL test was administered in children 6-11 years old to check psychopathological disorders.

**Results:**

Forty-four patients were included in the study. At the 1 year follow-up, “anxiety problems” (dependency, fears, worries, nervousness) developed in 47% of patients, “somatic problems” (aches, headaches, nausea, vomiting) in 29% and “affective problems” (crying, self-harming, worthlessness, guilt, tiredness, sadness) in 29%. Visual perception was statistically increased at the 1 year follow-up in our patient cohort (*p* = 0.0297), mainly among the meningitides group (*p* = 0.0189). Motor-coordination also increased at the follow-up check in the study participants (*p* = 0.0076), especially in the group affected by cerebellitis (*p* = 0.0302).

**Conclusions:**

Neuropsychological disorders are often difficult to recognize in the early stage. They must, however, be promptly identified through specific and standardized neuropsychological examinations in order to avoid long term sequelae in adulthood.

## Background

Acute central nervous system (ACNS) infections such as meningitis, encephalitis and cerebellitis still result in substantial morbidity and mortality despite the availability of effective antimicrobial therapy. Although physical and neurological complications, and hearing and visual impairments are most studied, neuropsychological sequelae and residual behavioral problems after ACNS infection have rarely been measured [1] Moreover, available studies have mainly investigated the school ability of children surviving meningitis, primarily through intellectual quotient (IQ), while just a few have focused on psychopathological impairment [2, 3].

In details, children surviving bacterial meningitis showed mildly decreased IQ and greatest impairment in verbal skills and organisational capacity than their classmates [4]. Published studies report academic and behavioural limitation in school-age survivors of meningitis [2, 5, 6]. As for the impact on quality-of-life, previous studies reported a lower score in meningitis than in the controls, especially concerning psychosocial health, cognition and family life [7–9].

Psychopathological impairment following ACNS infection may be classified in internalizing (ID) and externalizing disorders (ED) [10].

IDs are emotional and behavioral impairments, with high levels of negative affectivity. They include depressive disorders, anxiety disorders, and obsessive-compulsive and related disorders [11].

EDs are mental disorders characterized by maladaptive behavior directed toward an individual’s environment, causing impairment or interference in life functioning [12].

The objective of this study was to investigate whether children affected by ACNS infection develop any neuropsychological impairment measured with validated tests, at the 1 year follow-up.

In details the aim of our study is to find out if anxiety or other internalizing problems may affect ACNS infections survivors and if we can early detach them. In literature, meningitis has been associated with neurocognitive, educational, and psychological difficulties during childhood and early adolescence among survivors who are apparently healthy. In details, higher depressive and anxiety symptoms, psychological and behavioral problems, and increased risk of psychotic experiences have been reported after many years from the acute illness [13]. An early recognition of a psycopathological problem, whether possible, should be useful for preventing further disabilities.

## Methods

### Population and enrolment

We included all children aged 3-16 years old, consecutively admitted to the Bambino Gesù Children’s Hospital, Rome, Italy, for an ACNS infection from June 2013 to June 2015. Inclusion criteria were to be older than 3 years old at disease onset and not being affected by immunodeficiency, malignancy or underlying psychiatric pathology.

Participants were divided into three groups according to their final diagnosis of meningitis, cerebellitis and meningoencephalitis. According to the literature, acute cerebellitis was defined by clinical findings (ataxia, unsteady gait or fine motor movement, trembling of the head and trunk in an upright position and the extremities when attempting to move against gravity) [14] The diagnosis of meningitis, suspected on the base of clinical findings (such as fever, headache, vomiting and lethargy), was confirmed by the analysis of the cerebrospinal fluid, which typically revealed an elevated leukocyte count. Meningoencephalitis was diagnosed in case of a patient affected by meningitis and an altered level of consciousness and/or focal neurological abnormality, together with an abnormal electroencephalogram or neuroimaging findings and in absence of an alternative diagnosis [15].

During the defined study period, 84 children were hospitalized for ACNS at our hospital. Forty-four of these children were included in the study as they met all the inclusion criteria. The other 40 were excluded because they were younger than 3 years old at hospital admission. No death or physical sequelae was reported.

Group A included patients affected by meningitis (*n* = 13), group B those diagnosed with cerebellitis (*n* = 14) and group C those with meningoencephalitis (*n* = 17).

The children’s mean age was 6.4 years (range: 3.07- 16.45) and gender distribution was balanced (18 females and 26 males). During follow-up controls, patients also underwent neurological, hearing and visual examinations; none of these examinations showed any complications. Table 1 summarizes demographic data of patients.

**Table 1:** Demographic data of participants

	Group A		Group B		Group C		Total		*p*- value
	Meningitis		Cerebellitis		Meningoencephalitis				
Number of patients (n, %)	13	29.6	14	31.8	17	38.6	44	100.0	
Age, years (mean, SD)	7.0	3.3	6.2	2.6	6.1	3.8	6.4	3.3	0.694
Age < 6 years (n, %)	5	38.5	9	64.3	11	64.7	25	56.8	0.282
Sex male (n, %)	9	69.2	6	42.9	11	64.7	26	59.1	0.316
Race Caucasian (n, %)	12	92.3	14	100	17	100	43	97.7	0.295
Hospitalization,days (mean, SD)	14.2	5.7	9.1	4.1	26.7	20.5	49.9	113.5	0.002
Complications	0	0.0	0	0.0	3	17.6	3	6.8	0.078

Participants were divided into three groups according to their final diagnosis. Group A included patients affected by meningitis (*n* = 13), group B those diagnosed with cerebellitis (*n* = 14) and group C those with meningoencephalitis (*n* = 17). Table 1 summarizes demographic data of patients.

Both patients and their parents underwent a psychological interview during the first 2 weeks (from the 2nd to 13th days) of hospital admission and neuropsychological tests (T0). One year later, during a follow-up control, they repeated both the interview and the tests (T1).

The tests varied according to the patient’s age and ability to collaborate with the psychologist, who administered the tests, in English or Italian, and analyzed the results.

Informed consent was obtained from the patients’ parents.

### Neuropsychological tests

The following tests were administered to participants: the Leiter international performance scale – revised, the test of visual-motor integration, the child behavior checklist, the K-SADS-PL test.

#### Cognitive profile

The Leiter International Performance Scale – Revised (Leiter-R) is designed to be a non-verbal measure of intelligence and it consists of 20 sub-tests divided between two batteries: a Visualization and Reasoning Battery and an Attention and Memory Battery. The Leiter-R is for individuals between 2 years and 20 years 11 months of age. The Visualization and Reasoning Battery consists of 10 sub-tests in all; 4 sub-tests comprise a Brief IQ Screener for all ages, and two sets of 6 sub-tests are used to obtain a full scale intelligence quotient (FSIQ) [16].

#### Motor skills

The Developmental Test of Visual-Motor Integration (VMI) test is a neuropsychological test that analyzes visual construction skills. It identifies problems with visual perception, motor coordination, and visual-motor integration such as hand-eye coordination [17].

#### Psychopathological evaluation

The Child Behavior Checklist (CBCL M) is a widely used method of identifying problem behavior in children. Problems are identified by a respondent who knows the child well, usually a parent or other care giver. Alternative measures are available for teachers (the Teacher’s Report Form) and the child (the Youth Self Report). There are two versions of the checklist. The preschool checklist (CBCL/1½-5) is intended for use with children aged 18 months to 5 years. The school-age version (CBCL/6-18) is for children aged 6 to 18 years. It is an important measure for children’s emotional, behavioral and social aspects of life [18, 19].

The K-SADS-PL test is a diagnostic interview that can be administered in children between 6 and 11 years, and consists of a diagnostic interview for the evaluation of psychopathological disorders in children and adolescents. The theoretical model of Reference is essentially made up of the DSM-III-R and DSM-IV. The K-SADS-P is composed of: an unstructured introductory interview; a diagnostic screening interview that analyzes Primary symptoms of the diagnosis established in the test; an additional checklist; diagnostic supplements; a comprehensive checklist of the patient’s medical history; a scale for overall assessment of the child’s current functioning. It is administered by psychologists or child psychiatrists to both the children and their parents, and produces a score which takes into account the total of all the data collected from various sources available (family, children, teachers, pediatricians, etc.) [20].

#### Statistical analysis

Differences in demographic characteristics between the three groups of patients were studied through the Chi squared test or Fisher’s exact test for categorical variables, and F test (Anova) for the continuous variables. With regard to the neuro-psychological tests, after verifying that the differences in scores between T0 and T1 were not normally distributed, Wilcoxon signed rank tests for paired data were conducted to verify whether the cognitive level, constructive-praxic skills and psychopathological effects of children with meningitis, meningoencephalitis and cerebellitis measured at T0 differed with respect to those measured atT1. A *p*-value of ≤ .05 was considered statistically significant.

Stata 12 was used for the statistical analysis.

## Results

At T0, no neuropsychopathological impairment was detected in patients included in the study.

At the follow up, two patients dropped out from the study because their parents refused the follow-up controls: one belonged to group A and one to group B.

As for the others, ID disorders developed mainly in patients affected by meningitis and cerebellitis. Our results documented that participants with meningitis obtained higher scores at T1 than at T0 in the CBCL questionnaire: in particular, we observed a significant worsening in 84.62% of patients affected by meningitis (T0 mean score = 56.23; T1 mean score = 63.69; *p* = 0.0051). Similarly, 85.71% of children with cerebellitis had a significant worsening at the follow up (T0 mean score = 45.29; T1 mean score = 53.86; *p* = 0.0038).

In detail, the CBCL tests revealed newly diagnosed “anxiety problems” (dependency, fears, worries, nervousness) in 47% of patients, “somatic problems” (aches, headaches, nausea, vomiting) in 29% and “affective problems” (crying, self-harming, worthlessness, guilt, tiredness, sadness) in 29%.

**Fig. 1:**
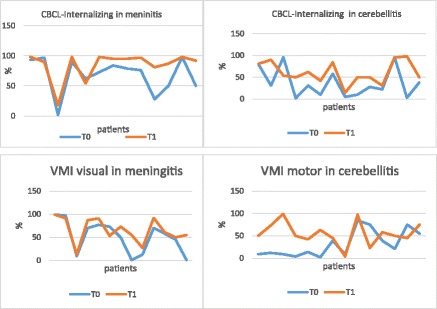
CBCL Internalizing and VMI in meningitis and cerebellitis at T0 and T1

Figure 1 summarizes the most important results, according to subgroups.

The Leiter International Performance Scale test performed at the disease onset and at the 1 year follow-up did not reveal statistically significant differences in the subgroups of patients. In particular, no impairment in QI was noted.

In the Visual perception test, the score increased significantly in 70.45% of patients at the 1 year follow-up. In detail, within the meningitides group the score increased in 84.62% of patients (T0 mean score = 99.23; T1 mean score = 108.15; *p* = 0.0189). Motor-coordination significantly increased at the follow-up check in 56.82% of the study participants. In particular, motor-coordination test values increased in 78.57% of patients affected by cerebellitis and the differences between T0 and T1 was statistically significant (T0 mean score = 90.36; T1 mean score = 103.14; *p* = 0.0302).

No statistically significant differences were noted for motor coordination, and visual-motor integration at the 1 year follow-up in patients who were affected by meningoencephalitis.

When considering age groups, of the ACNS infection survivors, 25 were younger than 6 years at diagnosis and 19 were older than 6 years. Neuropsychological outcome was worse in the youngest group. In detail, ID impairment was pathologic in 20% of cases in the younger children and in just 13% of children over 6 years of age. Comparing the CBCL results at T0 and T1, 72% of children younger than 6 years had a higher score at T1; in particular, the mean score was 49.8 at T0 and 55.84 at T1 (*p* = 0.0035). The VMI tests performed at disease onset and at the follow-up allowed us to identify problems with motor coordination in the youngest group. The increase was in 60% of patients aged less than 6 years, more specifically the mean score at T0 was 97.92 while at T1 was 105.76 (*p* = 0.0154).

In the older group, we noted a significant worsening of the CBCL ID results in 73.68% of patients: the mean score at T0 was 51.47, while at T1 was 55.53 (*p* = 0.0217). The visual perception test did not reveal statistically significant differences within each age group between T0 and T1.

**Fig. 2:**
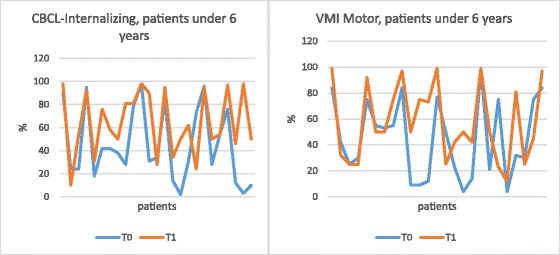
CBCL-Internalizing questionnaire and VMI motor test by age

Figure 2 summarizes the most important results, according to subgroups.

CBCL was used to recognize anxiety symptoms and not to make a diagnosis. We used this questionnaire instead of the KSADS interview due to the young age of the enrolled patients. Only semi-structured KSADs for the analysis of psychopathological aspects were administered to parents and children ages 6 to 11 years old. In the meningitis group, out of eight children, two children with anxiety disorders were found out. In the cerebellitis group, out of five children, a child revealed anxiety disturb and one deficit attention and hyperactivity. Out of the five children diagnosed with meningoencephalitis, one child was diagnosed with anxiety disorder.

## Discussion

Major sequelae of childhood ACNS, which include permanent neurologic disability, sensory and motor impairment, hearing and visual complications, and intellectual disability, have been studied extensively. Some studies reported that survivors of childhood ACNS infections are at increased risk of developmental, learning, and behavioral difficulties, even if they did not have acute neurologic complications or physical sequelae [21]. Other studies have stated that cognitive impairments are greater in those with a neurologic or physical disability, than those without (Christie D). In our study, no neurologic or physical disability has been reported at 1 year follow-up.

As for cognitive impairment, mild to severe intellectual disability is reported as a complication of bacterial meningitis in children. However, few studies have utilized appropriate controls and sufficient follow-up to assess the risk. Several studies have evaluated the IQ of survivors of bacterial meningitis compared with their siblings or other control children [22, 23]. In our study, we did not use a control group but we analyzed the same patients at enrollment and 1 year later. Consequently, the results should be more reliable. Moreover, in literature IQ WISC tests has been frequently used to calculate IQ [13]. We decided to evaluate IQ through the Leiter R test, in order to avoid the language development of children, and correlated age and race effects. Consequently, we collected data related to children under the age of 4 years, regardless of verbal abilities. Regardless of verbal abilities. We thus obtained an IQ evaluation also for the very young children who have less well-developed verbal skills in general and specifically an even more limited capacity to perfectly understand the Italian or English language.

We did not find any IQ impairment in any of our patients at the follow up examination. On the contrary, we did find ID impairment, mainly concerning anxiety.

Previous studies reported that ACNS’ survivors should be screened for emotional and behavioral difficulties, even if most patients fall within normal ranges on behavioral measures [8]. Identifying internalizing disorders may predict the risk of anxiety disorders in the future. Consequently, it is much important to psychologically monitor patients in order to prevent psychopathological disorders.

In our experience, psychopathological disorders must be promptly recognized in order to make every effort to help the patient and his/her family to deal with the problem. In particular, IQ impairment is difficult to check because patients tend to conceal their maladaptive emotions and cognitions, especially their anxiety status.

The understanding of internalizing disorders in young children has lagged behind advances in our understanding of other areas of psychopathology. One factor contributing to the relatively slower progress in this domain might be that, as a group, internalizing disorders tend to be viewed as less problematic by parents, teachers, and other caregivers. This may be related to the fact that such disorders are most often characterized by quiet, internal distress, rather than overtly, socially negative, or disruptive behavior [11].

A slight increase in internalizing symptoms had been previously reported in enterovirus ACNS survivors. This finding might be explained by central nervous system involvement in the brain areas related to emotional regulation [24].

Previous study evidenced survivors of childhood bacterial meningitis may suffer long-term disorders affecting the visual system and postural control [25]. For this reason, we used VMI test in order to analyze motor and visual ability in children even if they were hospitalized. In our study, we tested child’s ability of eye-hand coordination and fine-motor ability by the use of the appropriate sub-tests. Other studies have used the VMI test but did not use sub-tests [1, 6]. In this our sample, no significant differences have been detached at follow-up in motor and visual coordination skills.

Childhood psychological and behavioral problems are associated with an increased risk of depression in adult life [13]. Thus, individuals exposed to ACNS in childhood may have a high burden of neuropsychiatric disorders in adulthood.

Previous authors reported neuropsychological impairment several years after ACNS hospital admission.

In our report, we demonstrated that ID impairment can be diagnosed after a shorter follow-up, so that the patient can promptly undergo specific psychological controls.

Moreover, unlike previous reports in which only families/teachers were interviewed, the study relies on both parental reporting and childrens’ interviews [24]. The results are thus more reliable as the possibility of the parents over- or underestimating the severity of the psychological problem is reduced.

Finally, many studies have used short follow-ups; tests administered at the time of discharge from hospital may not reflect long-term neurocognitive performance. A psychological follow-up is mandatory to accurately measure the associations between ACNS and neuropsychological disabilities, possibly with the same psychologist in order to obtain the trust of the patient and the family.

The finding that younger age was linked to poorer outcome is contradictory to prior notions of brain plasticity (Ward 1942), but this suggests that the younger brain may be more vulnerable to brain insults caused by infectious critical illness.

## Conclusion

ID disorders, namely anxiety, somatic and affective problems, may develop early in ACNS infection survivors. They are difficult to recognize, as specific tests (CBCL1,5-5 and 6-18) are required, but they must be promptly identified in order to avoid depression in adult life.

CBCL was used to recognize anxiety symptoms and not may use to make a diagnosis. We used these questionnaire instead of the KSADS interview due to the young age of the enrolled patients.

Short-term follow-up and intervention is strongly recommended as what may initially present as subtle changes in cognitive function may, if left untreated, lead to long-lasting permanent disabilities [26].

The strength of our study is that it is a prospective study, with well-defined inclusion criteria and a fixed follow-up time, so that the bias inherent in the control study design is avoided. Observer bias was minimized through collection of data by a single dedicated researcher. Moreover, the use of standardized and validated measures of neuropsychological function and the close concordance between children and parent interviews confirms the relevance of the examinations. Furthermore, we evaluated IQ through the Leiter R test, so that we avoided the language development of children, and correlated age and race effects. In this way, we obtained an IQ evaluation also for very young children who have less well-developed verbal skills in general and specifically an even more limited capacity to perfectly understand the Italian or English language.

The study has some limitations. The first limit concerns the age of patients, which wides from 3 to 16 years. For this reason, the available tests were limited. In this context, CBCL questionnaire was the only one that can be equally administered to all participants. MASC and CDI questionnaires can be considered useful tools to detach anxiety and depressive symptoms, but can be used only in children over 8 years old. The VMI measures the extent to which individuals can integrate their visual and motor abilities. It is commonly used to identify children who are having significant difficulty with visual-motor integration and to determine the most appropriate course of action. The VMI is suitable for respondents with diverse environmental, educational, and linguistic backgrounds. Using only VMI is not sufficient to perform a complete neuropsychological evaluation and methodologically. Another tests exist for a better neuropsychological analysis; ABC Movement is an instrument that identifies and describes the difficulties of movement of children and adolescents from 3 to 16 years of age, but it was not possible to use the child’s health conditions during the hospitalization and the long timing of administration.

An other limit of our study is the small sample size, which did not allow as to divide the patients in subgroups according to the etiology, the clinical course and intensity. Finally, no longer follow-up had been performed at present. Patients will continue their clinical and neuropsychological controls.

Further studies are consequently necessary to validate our results.

## Abbreviations

ACNS: Acute central nervous system
CBCL: Child Behavior Checklist
ED: Externalizing disorders
ID: Internalizing disorders
Leiter-R: Leiter International Performance Scale – Revised
VMI: Visual-Motor Integration
