# Correction to: Neuropsychological and internalizing problems in acute central nervous system infections: a 1 year follow-up

**DOI:** 10.1186/s13052-017-0431-3

**Published:** 2017-12-27

**Authors:** Elena Bozzola, Paola Bergonzini, Mauro Bozzola, Alberto Eugenio Tozzi, Marco Masci, Chiara Rossetti, Emanuela Carloni, Alberto Villani

**Affiliations:** 1 Pediatric and Infectious Diseases Unit, IRCCS Bambino Gesù Children Hospital, Rome, Italy; 2 Internal Medicine and Therapeutics Department, Pediatrics and Adolescentology Unit, University of Pavia, Fondazione IRCCS San Matteo, Pavia, Italy; 3 Sanitary Direction, IRCCS Bambino Gesù Children Hospital, Rome, Italy

## Correction

The original article [1] contained an error mistakenly carried forward by the Production department handling this article whereby all authors’ names were incorrectly inverted.

The original article has now been corrected to reflect the correct names of all authors.
